# Safety and Feasibility of a CT‐Based Pathway for Left Atrial Appendage Assessment Prior to Inpatient Cardioversion: A Single‐Center Experience

**DOI:** 10.1002/clc.70323

**Published:** 2026-04-27

**Authors:** Ariel Furer, Maxim Perelman, Yafim Brodov, Orly Goitein, Sagit Ben Zikri, Rafael Kuperstein, Edward Itelman, Elad Maor, Eyal Nof, Roy Beinart, Shlomi Matetzky, Roy Beigel

**Affiliations:** ^1^ Department of Cardiology Chaim Sheba Medical Center Ramat‐Gan Israel; ^2^ Department of Military Medicine, Faculty of Medicine The Hebrew University of Jerusalem Jerusalem Israel; ^3^ Internal Medicine “T” Chaim Sheba Medical Center Ramat‐Gan Israel; ^4^ Gray Faculty of Medicine Tel Aviv University Tel Aviv Israel; ^5^ Department of Diagnostic Imaging Chaim Sheba Medical Center Ramat‐Gan Israel; ^6^ Cardiology Division Rabin Medical Center, Beilenson Campus Peta‐Tiqva Israel; ^7^ Arrhythmia Center Chaim Sheba Medical Center Ramat‐Gan Israel

**Keywords:** atrial fibrillation, cardioversion, computed tomography (CT), left atrial appendage, transesophageal echo

## Abstract

**Background:**

Transesophageal echocardiography (TEE) for left atrial appendage (LAA) thrombus exclusion before cardioversion faces logistical barriers including limited availability, patient intolerance, and procedural delays. We evaluated the feasibility and short‐term safety of a cardiac CT‐based alternative pathway in hospitalized patients with atrial fibrillation (AF).

**Methods:**

This retrospective single‐center study included 408 consecutive AF inpatients planned for cardioversion. The primary endpoint was the composite of stroke/TIA or systemic embolism within 48 h post‐cardioversion. Secondary endpoints included 30‐day thromboembolic events, acute kidney injury, and all‐cause mortality at 30 days and 1 year. Imaging modality selection was based on availability, clinical factors, and contraindications.

**Results:**

Among 74 patients undergoing CT‐guided cardioversion and 206 undergoing TEE‐guided cardioversion, no strokes or TIAs occurred within 48 h in either group (0/74 CT vs. 0/206 TEE, *p* = 1.0). By 30 days, one TIA occurred in the CT group (1.4%) and none in the TEE group (0%), representing an absolute risk difference of +1.4% (95% CI: −0.8% to +7.3%; *p* = 0.26). Acute kidney injury occurred in 8.1% of CT patients compared with 3.9% in the TEE group (absolute risk difference +4.2%, 95% CI: −1.4% to +12.9%; OR 2.18, 95% CI: 0.60–7.45; *p* = 0.21). No mortality was observed at 30 days or 1 year.

**Conclusions:**

In this single‐center feasibility analysis, a CT‐based imaging pathway for LAA assessment prior to cardioversion was associated with no periprocedural thromboembolic events and acceptable short‐term safety. Prospective randomized trials are needed to define the clinical role of CT as an alternative to TEE.

## Graphical abstract

An alternative for TEE? Cardiac CT is a practical alternative for ruling out LA/LAA thrombi in hospitalized patients who need cardioversion.

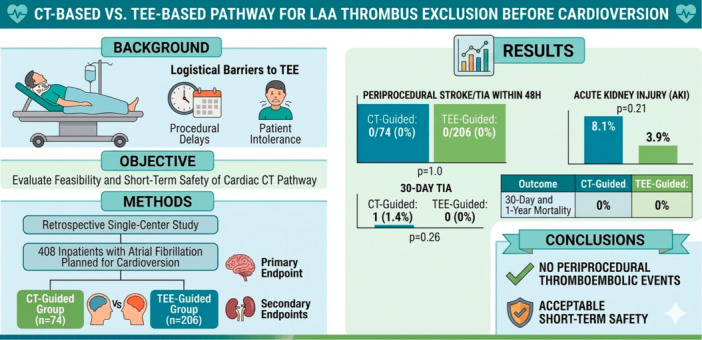

**Abbreviations**
AF: atrial fibrillation
CT: computed tomography
CVA: cerebrovascular accident
LA: left atrium
LAA: left atrial appendage
PVI: pulmonary vein isolation
TEE: transesophageal echocardiography

## 1 Introduction

Atrial fibrillation (AF) has emerged as a growing global health concern and is now widely recognized as a modern epidemic. It currently affects approximately 1%−2% of the general population [1], with recent reports suggesting this is an underestimation [2]. AF prevalence is rising sharply with age—particularly among individuals over the age of 80, where it is nearly eight times more common compared to younger populations [3, 4]. This trend is expected to continue and even accelerate in the coming decades, largely driven by the aging of populations worldwide [5]. In parallel, the increasing prevalence of modifiable and non‐modifiable risk factors—such as obesity, hypertension (HTN), diabetes, and heart failure—further contributes to the anticipated rise in AF incidence [6].

The management of AF is multifaceted and must be tailored to each patient's clinical profile and symptom burden. Broadly, therapeutic strategies can be categorized into two main approaches: rate control and rhythm control [7]. These may involve pharmacological or electrical cardioversion, as well as interventional procedures such as catheter ablation—most commonly pulmonary vein isolation (PVI)—which targets the triggers and substrates within the left atrium (LA) responsible for initiating and sustaining AF [8].

One of the major concerns preceding any of the interventions is the potential presence of thrombus within the LA, particularly in the left atrial appendage (LAA), where thrombi are detected in up to 10% of patients with AF. This poses a significant risk for embolic events, especially ischemic stroke, following cardioversion [9]. Consequently, current clinical guidelines recommend a minimum of 3 weeks of therapeutic anticoagulation prior to any intervention aimed at restoring sinus rhythm in patients with AF of more than 48 h duration or of unknown onset [7, 10]. Alternatively, if urgent cardioversion is being considered, transesophageal echocardiography (TEE) or other advanced imaging modalities can be used to assess for the presence of thrombus in the LA and particularly the LAA. If imaging confirms the absence of thrombus, cardioversion may proceed safely without the full 3‐week course of anticoagulation [11, 12].

The most widely utilized imaging modality in this clinical context is TEE, which is considered the gold standard for excluding thrombus in the LA and particularly the LAA [13]. However, due to growing clinical demand and the limited availability and semi‐invasive nature of TEE, cardiac computed tomography (CT) has gained traction over the past two decades as a viable noninvasive alternative for thrombus detection in AF patients [14]. Early studies demonstrated that CT achieves a sensitivity of 100% for detecting LA/LAA thrombi when compared with TEE. Nonetheless, initial concerns were raised regarding its relatively low and variable positive predictive value (PPV), which ranged between 41% and 92% depending on the timing and protocol of image acquisition [15]. More recent investigations, however, have addressed these limitations by incorporating delayed contrast‐enhanced imaging protocols, which have substantially improved diagnostic accuracy [16]. With these advancements, studies have reported PPV and specificity approaching 100%, positioning CT as a promising tool for pre‐cardioversion thrombus assessment when TEE is not feasible or readily available [17].

In the pre‐procedural evaluation for PVI, cardiac CT has become an essential component and is now the most commonly employed imaging modality. It serves a dual role: providing high‐resolution anatomical visualization of the LA and pulmonary veins, and excluding the presence of thrombus in the LA, particularly within the LAA [18]. Despite its growing use in the PVI setting, there remains a significant gap in the literature regarding the real‐world application of CT compared to TEE in patients undergoing cardioversion—whether electrical or pharmacologic [19].

Most of the meta‐analyses comparing CT and TEE for LAA thrombus detection have focused almost exclusively on pre‐ablation patient populations [15, 20]. Notably, only one small‐scale study from 2004 included patients undergoing cardioversion, analyzing 52 consecutive cases. As such, current evidence on the role of CT in this specific context remains limited [21].

The 2023 joint guidelines from the American College of Cardiology (ACC), American Heart Association (AHA), American College of Chest Physicians (ACCP), and Heart Rhythm Society (HRS) acknowledge CT as a viable alternative imaging modality for the diagnosis of device‐related thrombosis, particularly in patients who have undergone LAA occlusion [7]. However, they stopped short of formally recommending CT for LAA thrombus exclusion prior to cardioversion. The 2024 European Society of Cardiology (ESC) Guidelines for the management of AF provide more definitive recommendations for pre‐cardioversion imaging. They endorse TEE as the standard of care for excluding LA/LAA thrombus in this setting, while CT is primarily recommended for anatomical assessment in preparation for interventions such as PVI [10].

Given these practical challenges with TEE‐based screening, we sought to evaluate the feasibility and short‐term clinical safety of implementing a cardiac CT‐based pathway for LAA assessment in hospitalized patients requiring urgent cardioversion. This study was designed as a care‐pathway evaluation rather than a head‐to‐head diagnostic accuracy comparison, with the primary objective of assessing 48 h thromboembolic event rates following CT‐guided versus TEE‐guided cardioversion at our institution.

## 2 Materials and Methods

### 2.1 Study Design

This study was a retrospective, single‐center analysis conducted at the Sheba Medical Center. We reviewed the medical records of all consecutive patients admitted to the cardiology ward who underwent cardioversion for AF between September 1, 2014, and November 30, 2022. Each case was evaluated individually to determine the imaging modality—either TEE or cardiac CT—that was used as part of the pre‐cardioversion workup to assess for the presence of thrombus in the LA/LAA.

Comprehensive clinical and laboratory data were collected through the hospital's electronic medical record system. The data set included demographic information such as age, sex, and body mass index (BMI), as well as relevant comorbidities. These included HTN, diabetes mellitus (DM), history of cerebrovascular accident (CVA) or transient ischemic attack (TIA), and chronic kidney disease (CKD). Additionally, we recorded the use and status of anticoagulation therapy prior to cardioversion.

Thromboembolic risk stratification was performed using the CHA₂DS₂‐VASc score for each patient based on documented comorbidities in the electronic medical record. All components were ascertained through a systematic review of electronic medical records including discharge diagnoses, cardiology notes, echocardiographic reports, and prior imaging studies [22].

The inclusion criteria for this study required patients to be 18 years of age or older, to have undergone at least one imaging modality—either TEE or cardiac CT—within 48 h prior to cardioversion, and to have had at least one documented attempt at electrical cardioversion during their hospitalization.

Following cohort identification, we conducted a comparative analysis between patients who underwent TEE and those who underwent cardiac CT as the pre‐cardioversion imaging modality for exclusion of thrombus in the LA/LAA. This analysis aimed to evaluate differences in patient characteristics, imaging utilization patterns, and clinical context for modality selection.

Patients in whom CT suggested the presence of LAA thrombus did not undergo confirmatory TEE and did not proceed to cardioversion (verification bias). These patients were managed with rate control and anticoagulation for at least 3 weeks before repeat imaging was considered. This study design reflects real‐world clinical practice but limits our ability to directly compare the diagnostic accuracy of the two modalities.

### 2.2 Study Endpoints

The prespecified primary safety endpoint was the composite of acute ischemic stroke, TIA, or systemic embolism occurring within 48 h of cardioversion, representing immediate periprocedural thromboembolic risk. Secondary endpoints included stroke/TIA at 30 days, all‐cause mortality at 30 days and 1 year, and post‐procedural acute kidney injury (AKI).

AKI was defined according to KDIGO criteria as an increase in serum creatinine of ≥ 0.3 mg/dL within 48 h of contrast administration or ≥ 1.5 times the baseline value within 7 days. Baseline creatinine was defined as the most recent value measured prior to imaging during the same hospitalization. Thromboembolic events were identified based on new diagnostic documentation within 30 days of cardioversion, validated through review of clinical notes, neurology consultations, imaging reports, and discharge summaries. Mortality data were sourced from the Israeli National Population Registry and the hospital's electronic medical records. All outcome data were extracted from patients' electronic medical records.

### 2.3 Imaging Modalities

#### 2.3.1 TEE

TEE was performed for the purpose of excluding thrombus in the LA and LAA. All TEE examinations were conducted by board‐certified cardiologists with subspecialty training in echocardiography. Procedures were carried out using a standardized protocol that included mild conscious sedation, typically achieved with intravenous administration of midazolam, to ensure patient comfort and procedural tolerance. High‐quality two‐dimensional and Doppler imaging was obtained in multiple views to allow thorough visualization of the LAA.

#### 2.3.2 Cardiac CT

Cardiac CT studies were performed using 256‐slice multidetector CT scanners. Across the study period (2014–2022), two platforms were used: the Brilliance iCT (Philips Healthcare, Cleveland, OH, USA) for the majority of studies and the Revolution CT (GE Healthcare) for a subset; the detailed acquisition parameters below reflect the Philips protocol. Since cardiac anatomy rather than coronary imaging was the indication, no β‐blockers were administered for heart rate reduction. Early‐phase scanning was initiated using a bolus‐tracking technique with a region of interest placed in the ascending aorta, triggering at a threshold of 100 Hounsfield units above baseline. Early‐phase acquisition parameters were: voltage 100–120 kV, effective tube current 800–1235 mAs, slice collimation 256 × 0.625 mm, gantry rotation time 270 ms, pitch 0.2, and table feed 3.3 mm/rotation; the scan was acquired in a single breath‐hold with retrospective ECG gating and reconstructed at a systolic phase of 30% or 40% of the R–R interval depending on image quality. Contrast medium consisted of 80 mL of non‐ionic iodinated contrast (Iomeron 350, Bracco Imaging, Milano, Italy) administered intravenously at a flow rate of 4–5 mL/s. A late‐phase scan was performed 45 s after completion of the early‐phase acquisition using reduced parameters (30 × 0.6 mm collimation, 120 kV, 200 mAs) covering the aortic arch to the mid‐left ventricle. Average radiation exposure was: dose length product 900 ± 200 mGy·cm. Images were analyzed on a dedicated workstation (Extended Brilliance Workspace version 4.5, Philips Healthcare). On CT, a thrombus was defined as a filling defect present on both early‐ and late‐phase images with an oval or round morphology on the late phase; circulatory stasis was defined as a triangular filling defect in the LAA visible on the early phase only and absent on the late‐phase image [23, 24]. Three experienced cardiovascular radiologists independently reviewed all images; in cases of disagreement, a consensus was reached by joint reading. All readers had more than 10 years of expertise in cardiac imaging. Formal inter‐reader agreement statistics were not prospectively collected in this retrospective study.

For each individual patient, data related to thrombus detection were extracted from the electronic medical records. This included documentation from radiology and echocardiography reports, cardiologist interpretations, and procedural notes detailing the presence or absence of thrombus in the LA/LAA.

The choice of imaging modality—TEE or cardiac CT—was determined by clinical staff based on several factors. These included the availability of the imaging modality within 24 h of the planned cardioversion procedure, as well as patient‐specific considerations such as absolute or relative contraindications.

### 2.4 Statistical Analysis

Continuous variables were summarized as mean ± standard deviation (SD) for normally distributed data or as median with interquartile range (IQR) for non‐normally distributed data. The normality of continuous variables was assessed using the Anderson−Darling and Shapiro−Wilk tests. Categorical variables were presented as absolute counts with corresponding percentages.

For comparisons between groups, continuous variables were analyzed using the Student's *t*‐test for normally distributed data. All binary outcome comparisons were performed using the two‐sided Fisher's exact test, applied to all dichotomous endpoints including thromboembolic events, AKI, composite outcomes, and mortality. Between‐group risk differences (CT minus TEE) were calculated with 95% confidence intervals using the Wilson score‐based method described by Newcombe. Odds ratios (OR) with exact conditional 95% confidence intervals were derived from the hypergeometric distribution using the conditional maximum likelihood approach. When one group had zero events, the OR could not be reliably estimated and is not reported.

All statistical analyses were conducted using R software, version 4.1.0 (R Foundation for Statistical Computing, Vienna, Austria). A two‐tailed *p* < 0.05 was considered statistically significant.

## 3 Results

**Figure 1:**
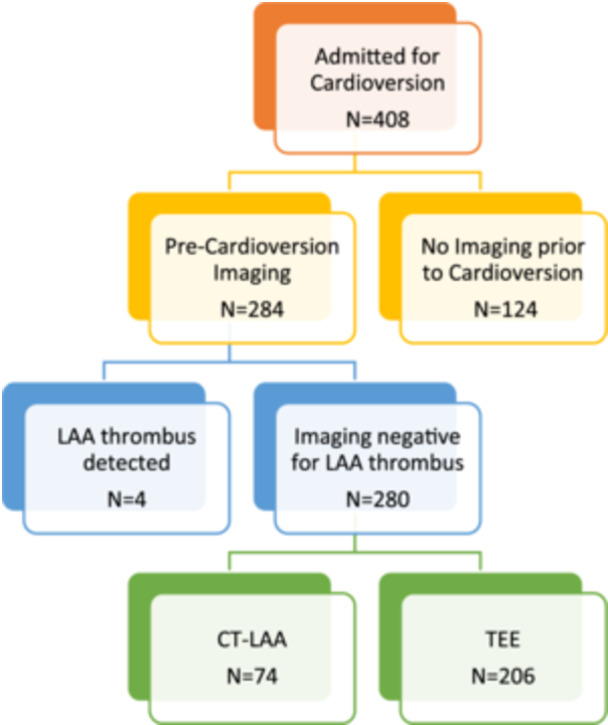
Schematic flowchart of the study. Schematic visualization illustrating the inclusion and exclusion process for the final analysis, comparing two groups of patients admitted to the cardiology department for cardioversion. These patients underwent pre‐cardioversion imaging using either cardiac CT or TEE. CT, computed tomography; TEE, transesophageal echocardiography.

During the study period, the records of 408 consecutive patients who were admitted and intended to undergo electrical cardioversion for AF were evaluated. Of these, 124 patients (30.4%) did not undergo pre‐cardioversion imaging and were excluded from analysis; baseline characteristics of this cohort are presented in Table S1. Among the remaining 284 patients who underwent imaging, four had LAA thrombus identified on pre‐cardioversion evaluation (three in the CT group and one in the TEE group) and did not proceed to cardioversion; these patients were managed with rate control and anticoagulation for at least 3 weeks before additional imaging was considered. The final outcomes analysis, therefore, comprised 280 patients who underwent cardioversion following negative imaging: 74 (26%) evaluated with CT and 206 (74%) evaluated with TEE (Figure 1).

### 3.1 Baseline Characteristics

The median age was 69 years (IQR, 61.0−76.0), with 174 males (62%), and the median BMI was 28 (IQR, 25−31). In addition, 183 (65%) suffered from HTN, 86 (31%) had DM, 51 (18%) had CKD, and 32 (11%) were diagnosed with CVA/TIA prior to enrollment. Patients' baseline demographic and medical characteristics are shown in Table 1 as detailed. We found no significant differences between the two groups regarding their baseline characteristics. Anticoagulation characteristics are detailed in Table S2.

**Table 1:** Baseline characteristics of the entire cohort and comparison between the group of patients who had undergone computed tomography for evaluation of the left atrium and left atrial appendage (CT‐LAA) and those who had undergone transesophageal echocardiography.

	Overall	CT‐LAA	TEE	*p* value
Number of patients (%)	280	74 (26.4)	206 (73.6)	
Median age (IQR, 25%−75%)	69.0 (61−76)	67 (59.2−74)	69 (62−77)	0.06
Sex (males, *N* [%]))	174 (62.1)	39 (52.7)	135 (65.5)	0.05
Median BMI (IQR, 25%−75%)	28 (25−31)	28 (25−31)	28 (25−32)	0.88
HTN (*N* [%])	183 (65.4)	46 (62.2)	137 (66.5)	0.50
DM (*N* [%])	86 (30.7)	21 (28.4)	65 (31.6)	0.61
Prior CVA\TIA (*N* [%])	32 (11.4)	9 (12.2)	23 (11.2)	0.82
Median CHA₂DS₂‐VASc score (IQR, 25%–75%)	3.0 (2–5)	3.0 (1–5)	3.0 (2–5)	0.34
CHA₂DS₂‐VASc ≥ 2, *N* (%)	216 (77.1%)	52 (70.3%)	164 (79.6%)	0.10
Prior CKD (*N* [%])	51 (18.2)	9 (12.2)	42 (20.4)	0.12
Prior anticoagulation (*N* [%])	278 (99.3)	74 (100)	204 (99.0)	0.39

*Note:* CHA₂DS₂‐VASc score calculated using the full standard nine‐component algorithm: congestive heart failure (1 point), hypertension (1 point), age ≥ 75 years (2 points), diabetes mellitus (1 point), prior stroke/TIA/thromboembolism (2 points), vascular disease including coronary artery disease, prior myocardial infarction, or peripheral arterial disease (1 point), age 65–74 years (1 point), and female sex (1 point); maximum possible score 9 points.

Abbreviations: BMI, body mass index; CKD, chronic kidney disease; CT‐LAA, computed tomography aimed to visualize left atrial appendage; CVA, cerebrovascular accident; DM, diabetes mellitus; HTN, hypertension; TEE, transesophageal echocardiography; TIA, transient ischemic event.

### 3.2 Outcomes

As detailed in Table 2, for the prespecified primary safety endpoint, no strokes, TIAs, or systemic embolic events occurred within 48 h of cardioversion in either the CT‐guided group (0/74, 0%) or the TEE‐guided group (0/206, 0%) (Fisher's exact test, *p* = 1.0).

At 30 days post‐cardioversion, one TIA event occurred in the CT group (1/74, 1.4%) and none in the TEE group (0/206, 0%), representing an absolute risk difference of +1.4% (95% CI: −0.8% to +7.3%; Fisher's exact test, *p* = 0.26). The OR could not be reliably estimated due to zero events in the TEE group.

No deaths occurred in either the CT group (0/74) or the TEE group (0/206) at 30‐day or 1‐year follow‐up. AKI occurred in 6/74 (8.1%) patients in the CT group compared with 8/206 (3.9%) in the TEE group, representing an absolute risk difference of +4.2% (95% CI: −1.4% to +12.9%; Fisher's exact test, *p* = 0.21) and an OR of 2.18 (95% CI: 0.60–7.45). The composite outcome of AKI or stroke/TIA occurred in 7/74 (9.5%) patients in the CT group compared with 8/206 (3.9%) in the TEE group (absolute risk difference +5.6%, 95% CI: −0.4% to +14.6%; OR 2.58, 95% CI: 0.76–8.48; Fisher's exact test, *p* = 0.08).

**Table 2:** Clinical outcomes following CT‐guided versus TEE‐guided cardioversion.

Outcome	Overall (*N* = 280)	CT‐LAA (*N* = 74)	TEE (*N* = 206)	*p* value^a^	Effect size (95% CI)
*Thromboembolic events*					
CVA/TIA at 48 h, *N* (%)	0 (0%)	0 (0%)	0 (0%)	1.0	OR not estimable^b^
CVA/TIA at 30 days, *N* (%) RD + 1.4% (95% CI −0.8% to +7.3%)	1 (0.4%)	1 (1.4%)	0 (0%)	0.26	OR not estimable^b^
*Mortality*					
All‐cause mortality at 30 days, *N* (%)	0 (0%)	0 (0%)	0 (0%)	1.0	—
All‐cause mortality at 1 year, *N* (%)	0 (0%)	0 (0%)	0 (0%)	1.0	—
*Renal outcomes*					
Acute kidney injury, *N* (%) RD + 4.2% (95% CI −1.4% to +12.9%)	14 (5.0%)	6 (8.1%)	8 (3.9%)	0.21	OR 2.18 (0.60–7.45)
*Composite outcome*					
AKI or CVA/TIA, *N* (%) RD + 5.6% (95% CI −0.4% to +14.6%)	15 (5.4%)	7 (9.5%)	8 (3.9%)	0.08	OR 2.58 (0.76–8.48)

Abbreviations: AKI, acute kidney injury (defined per KDIGO criteria as serum creatinine increase ≥ 0.3 mg/dL within 48 h or ≥ 1.5× baseline within 7 days of contrast administration); CI, confidence interval; CVA, cerebrovascular accident; CT‐LAA, computed tomography–left atrial appendage; OR, odds ratio; RD, risk difference; TEE, transesophageal echocardiography; TIA, transient ischemic attack.

a All *p* values from two‐sided Fisher's exact test.

b OR could not be estimated due to zero events in the TEE group.

### 3.3 Statistical Power and Effect Size Interpretation

Given the observed event rates and sample sizes (*n* = 74 CT, *n* = 206 TEE), this study was substantially underpowered to detect clinically meaningful differences in outcomes. Assuming a baseline 30‐day stroke/TIA rate of 1.5% (as observed in the TEE group) and 80% power with *α* = 0.05, detecting a clinically relevant absolute risk difference of 2% would require approximately 956 patients per group. For the AKI endpoint, with the observed rates of 8.1% and 3.9%, 80% power would require approximately 495 patients per group; our study had an estimated post hoc power of 18.9% for this comparison.

## 4 Discussion

In the current study, which evaluated a CT‐based pathway for LA/LAA thrombus assessment prior to cardioversion in hospitalized patients with AF, we observed no periprocedural thromboembolic events in either group and similar rates of adverse events at 30 days. While these preliminary findings suggest CT‐based pathways may be feasible in selected patients, our study design precludes definitive conclusions regarding comparative safety or equivalence to TEE.

Our experience describes the real‐world implementation of CT for LA/LAA thrombus assessment prior to cardioversion. CT offers several practical advantages over TEE: it is noninvasive, does not require sedation, is less operator‐dependent, and may be more readily available given the global shortage of skilled echocardiographers. Contemporary studies comparing CT and TEE report similar sensitivity for thrombus detection, with delayed‐phase imaging protocols achieving PPV approaching those of TEE [15, 20, 25, 26, 27].

While CT effectively excludes LAA thrombus and provides anatomical visualization, it is important to recognize that CT alone does not comprehensively phenotype atrial substrate or cardiomyopathy. Contemporary assessment of atrial cardiomyopathy is inherently multimodal, incorporating echocardiographic functional parameters (e.g., left atrial strain), cardiovascular magnetic resonance tissue characterization (e.g., atrial fibrosis quantification), electrocardiographic indices (e.g., P‐wave duration, terminal force), circulating biomarkers (e.g., NT‐proBNP, fibrosis markers), and invasive electroanatomic voltage mapping. CT serves a complementary role in this multimodal framework, primarily for anatomical delineation and thrombus exclusion rather than as a stand‐alone solution for atrial substrate characterization [28].

Our primary aim was to evaluate the safety profile of CT compared to TEE in a real‐world cardioversion population. Four patients (three CT, one TEE) had LAA thrombus detected and did not proceed to cardioversion, instead receiving rate control and extended anticoagulation. Among the remaining patients who underwent cardioversion, both imaging groups demonstrated low rates of adverse events including stroke and mortality. The incidence of AKI, however, warrants careful consideration. AKI occurred in 8.1% of CT patients versus 3.9% of TEE patients (OR 2.18, 95% CI 0.60−7.45, *p* = 0.21). While not statistically significant and consistent with contemporary evidence that modern low‐osmolality contrast carries acceptable risk in patients with preserved renal function [29, 30, 31], this numerically higher rate warrants cautious interpretation, particularly given our broad 7‐day AKI definition.

Anticoagulation quality warrants consideration as a potential confounder. Among the 48 patients (17.1%) receiving vitamin K antagonist therapy, therapeutic INR control (2.0–3.0) was achieved in only 54.2% overall, with particularly low rates in the CT group (28.6%) compared to the TEE group (58.5%), though this difference did not reach statistical significance due to small numbers (*p* = 0.23). This finding likely reflects that CT patients represented more urgent cases with shorter, less established anticoagulation duration—consistent with the higher proportion of CT patients with less than 48 h of pre‐cardioversion anticoagulation (58.1% vs. 51.0% TEE).

Recently, the LACLOT [32] randomized controlled trial compared CT and TEE as pre‐cardioversion imaging modalities for detecting left atrial thrombus in 102 hospitalized AF patients. The study found that CT significantly reduced time to imaging (7.1 vs. 24.3 h) and time to cardioversion (1.7 vs. 4.6 days), while also improving quality of life scores at discharge compared to TEE. Moreover, there were no significant differences between groups in adverse events, including stroke, embolism, or AKI, which is in line with our results. The LACLOT study results also support CT as a viable, efficient, and patient‐friendly alternative to TEE for thrombus exclusion prior to cardioversion.

### 4.1 Limitations

This study has several important limitations. As a retrospective, single‐center analysis, imaging modality selection was determined by clinical availability and patient‐specific factors rather than randomization, introducing selection bias and confounding by indication that cannot be fully addressed through statistical adjustment. Multivariable adjustment and propensity score methods were precluded by the small number of outcome events. Crude comparisons should therefore be interpreted with caution and cannot support causal inferences. Additionally, patients with CT‐detected thrombus did not undergo confirmatory TEE and were excluded from cardioversion (verification bias), potentially favoring the observed safety profile of the CT pathway.

Our study was underpowered to detect clinically meaningful differences in outcomes. With 74 CT and 206 TEE patients and rare events, we could detect only large differences (6%−8% absolute risk); clinically relevant differences of 1%−2% would require approximately 956 patients per group for the primary endpoint and 495 for the AKI endpoint. The wide confidence intervals include both potential benefit and harm, precluding conclusions about equivalence or non‐inferiority.

Although our 7‐day creatinine follow‐up window aligns with KDIGO criteria for contrast‐associated AKI, it may still capture renal injury attributable to factors other than contrast exposure in this hospitalized cohort. AKI staging data (KDIGO Stages 1–3) were not systematically collected in this retrospective study. We also lacked complete data on baseline renal function and anticoagulation quality metrics for all patients.

These findings should be interpreted as hypothesis‐generating, demonstrating feasibility but not establishing equivalence to TEE.

## 5 Conclusion

In this single‐center feasibility analysis, a CT‐based imaging pathway for LAA assessment prior to cardioversion was associated with no periprocedural thromboembolic events and acceptable short‐term safety outcomes in selected hospitalized patients. While these preliminary findings are encouraging, our retrospective study design, verification bias, and insufficient statistical power preclude definitive conclusions regarding equivalence to TEE. Prospective randomized trials with adequate sample sizes are needed to establish the appropriate clinical role of CT as an alternative to TEE for pre‐cardioversion thrombus assessment in patients with AF.

## Author Contributions

Conceptualization: Roy Beigel, Ariel Furer, and Maxim Perelman. Methodology: Roy Beigel, Ariel Furer, and Maxim Perelman. Software: Maxim Perelman and Edward Itelman. Validation: Roy Beigel, Ariel Furer, and Maxim Perelman. Formal analysis: Maxim Perelman, Edward Itelman, and Ariel Furer. Investigation: Roy Beigel, Ariel Furer, Yafim Brodov, Orly Goitein, Sagit Ben Zikri, Rafael Kuperstein, and Maxim Perelman. Resources: Yafim Brodov, Orly Goitein, Sagit Ben Zikri, Rafael Kuperstein, Eyal Nof, Roy Beigel, and Shlomi Matetzky. Data curation: Elad Maor, Maxim Perelman, Ariel Furer, and Roy Beigel. Writing original draft preparation: Maxim Perelman, Ariel Furer, and Roy Beigel. Writing, review, and editing: Roy Beinart, Yafim Brodov, Orly Goitein, Sagit Ben Zikri, Rafael Kuperstein, Eyal Nof, Roy Beinart, and Shlomi Matetzky. Visualization: Maxim Perelman and Ariel Furer. Supervision: Roy Beigel. All authors have read and agreed to the published version of the manuscript.

## Funding

The authors have nothing to report.

## Ethics Statement

The study was conducted in accordance with the Declaration of Helsinki and approved by the Institutional Review Board of the Sheba Medical Center (SMC‐15‐2669).

## Consent

Patient consent was waived due to a retrospective study design based on deidentified data from the electronic medical record in alignment with the Institutional Review Board approval.

## Conflicts of Interest

The authors declare no conflicts of interest.

## Supporting information

Supporting File 1

Supporting File 2

## Data Availability

The data supporting the findings of this study are available from the corresponding author upon reasonable request. All authors had access to the data and a role in writing the manuscript.

## References

[1] J. Kornej , C. S. Börschel , E. J. Benjamin , and R. B. Schnabel , “Epidemiology of Atrial Fibrillation in the 21st Century: Novel Methods and New Insights,” Circulation Research 127, no. 1 (2020): 4–20, 10.1161/CIRCRESAHA.120.316340. 32716709 PMC7577553

[2] J. J. Noubiap , J. J. Tang , J. T. Teraoka , T. A. Dewland , and G. M. Marcus , “Minimum National Prevalence of Diagnosed Atrial Fibrillation Inferred From California Acute Care Facilities,” Journal of the American College of Cardiology 84, no. 15 (2024): 1501–1508, 10.1016/J.JACC.2024.07.014. 39269390

[3] M. Kavousi , “Differences in Epidemiology and Risk Factors for Atrial Fibrillation Between Women and Men,” Frontiers in Cardiovascular Medicine 7 (2020): 3, 10.3389/FCVM.2020.00003. 32118043 PMC7025483

[4] R. Sankaranarayanan , G. Kirkwood , K. Dibb , and C. J. Garratt , “Comparison of Atrial Fibrillation in the Young Versus That in the Elderly: A Review,” Cardiology Research and Practice 2013, no. 1 (2013): 976976, 10.1155/2013/976976. 23401843 PMC3564268

[5] K. Wasmer , L. Eckardt , and G. Breithardt , “Predisposing Factors for Atrial Fibrillation in the Elderly,” Journal of Geriatric Cardiology: JGC 14, no. 3 (2017): 179–184, 10.11909/J.ISSN.1671-5411.2017.03.010. 28592961 PMC5460064

[6] M. K. Chung , L. L. Eckhardt , L. Y. Chen , et al., “Lifestyle and Risk Factor Modification for Reduction of Atrial Fibrillation: A Scientific Statement From the American Heart Association,” Circulation 141, no. 16 (2020): E750–E772, 10.1161/CIR.0000000000000748/SUPPL_FILE/DATA. 32148086

[7] J. A. Joglar , M. K. Chung , A. L. Armbruster , et al., “2023 ACC/AHA/ACCP/HRS Guideline for the Diagnosis and Management of Atrial Fibrillation: A Report of the American College of Cardiology/American Heart Association Joint Committee on Clinical Practice Guidelines,” Circulation 149, no. 1 (2024): E1–E156, 10.1161/CIR.0000000000001193. 38033089 PMC11095842

[8] S. Zafeiropoulos , I. Doundoulakis , A. Bekiaridou , et al., “Rhythm vs Rate Control Strategy for Atrial Fibrillation,” JACC: Clinical Electrophysiology 10, no. 7 (2024): 1395–1405, 10.1016/j.jacep.2024.03.006. 38727662

[9] M. N. D. D. Minno , P. Ambrosino , A. D. Russo , M. Casella , E. Tremoli , and C. Tondo , “Prevalence of Left Atrial Thrombus in Patients With Non‐Valvular Atrial Fibrillation,” Thrombosis and Haemostasis 115, no. 03 (2016): 663–677. 26607276 10.1160/TH15-07-0532

[10] I. C. Van Gelder , M. Rienstra , K. V. Bunting , et al., “2024 ESC Guidelines for the Management of Atrial Fibrillation Developed in Collaboration With the European Association for Cardio‐Thoracic Surgery (EACTS),” European Heart Journal 45, no. 36 (2024): 3314–3414, 10.1093/EURHEARTJ/EHAE176. 39210723

[11] A. Brandes , H. J. G. M. Crijns , M. Rienstra , et al., “Cardioversion of Atrial Fibrillation and Atrial Flutter Revisited: Current Evidence and Practical Guidance for a Common Procedure,” EP Europace 22, no. 8 (2020): 1149–1161. 10.1093/europace/euaa057 PMC7399700 32337542

[12] G. F. Michaud and W. G. Stevenson , “Atrial Fibrillation,” New England Journal of Medicine 384, no. 4 (2021): 353–361, 10.1056/NEJMCP2023658. 33503344

[13] S. Yu , H. Zhang , and H. Li , “Cardiac Computed Tomography Versus Transesophageal Echocardiography for the Detection of Left Atrial Appendage Thrombus: A Systematic Review and Meta‐Analysis,” Journal of the American Heart Association 10, no. 23 (2021): e022505, 10.1161/JAHA.121.022505. 34796743 PMC9075398

[14] R. Beigel , N. C. Wunderlich , S. Y. Ho , R. Arsanjani , and R. J. Siegel , “The Left Atrial Appendage: Anatomy, Function, and Noninvasive Evaluation,” JACC: Cardiovascular Imaging 7, no. 12 (2014): 1251–1265, 10.1016/j.jcmg.2014.08.009. 25496544

[15] T. Vira , P. Pechlivanoglou , K. Connelly , H. C. Wijeysundera , and I. Roifman , “Cardiac Computed Tomography and Magnetic Resonance Imaging vs. Transoesophageal Echocardiography for Diagnosing Left Atrial Appendage Thrombi,” EP Europace 21, no. 1 (2019): e1–e10, 10.1093/EUROPACE/EUY142. 29961869

[16] P. Spagnolo , M. Giglio , D. Di Marco , et al., “Diagnosis of Left Atrial Appendage Thrombus in Patients With Atrial Fibrillation: Delayed Contrast‐Enhanced Cardiac CT,” European Radiology 31, no. 3 (2021): 1236–1244, 10.1007/S00330-020-07172-2. 32886202 PMC7880950

[17] R. A. Quintana , T. Dong , R. Vajapey , et al., “Preprocedural Multimodality Imaging in Atrial Fibrillation,” Circulation: Cardiovascular Imaging 15, no. 10 (2022): 733–746, 10.1161/CIRCIMAGING.122.014386. 36256725

[18] S. Liddy , U. Buckley , H. K. Kok , et al., “Applications of Cardiac Computed Tomography in Electrophysiology Intervention,” European Heart Journal‐Cardiovascular Imaging 19, no. 3 (2018): 253–261, 10.1093/EHJCI/JEX312. 29236953

[19] F. Pathan , H. Hecht , J. Narula , and T. H. Marwick , “Roles of Transesophageal Echocardiography and Cardiac Computed Tomography for Evaluation of Left Atrial Thrombus and Associated Pathology,” JACC. Cardiovascular Imaging 11, no. 4 (2018): 616–627, 10.1016/j.jcmg.2017.12.019. 29622180

[20] J. Romero , S. A. Husain , I. Kelesidis , J. Sanz , H. M. Medina , and M. J. Garcia , “Detection of Left Atrial Appendage Thrombus by Cardiac Computed Tomography in Patients With Atrial Fibrillation: A Meta‐Analysis,” Circulation: Cardiovascular Imaging 6, no. 2 (2013): 185–194, 10.1161/CIRCIMAGING.112.000153. 23406625

[21] S. Achenbach , D. Sacher , D. Ropers , et al., “Electron Beam Computed Tomography for the Detection of Left Atrial Thrombi in Patients With Atrial Fibrillation,” Heart 90, no. 12 (2004): 1477–1478, 10.1136/HRT.2003.027805. 15547034 PMC1768585

[22] G. Y. H. Lip , R. Nieuwlaat , R. Pisters , D. A. Lane , and H. J. G. M. Crijns , “Refining Clinical Risk Stratification for Predicting Stroke and Thromboembolism in Atrial Fibrillation Using a Novel Risk Factor‐Based Approach,” Chest 137, no. 2 (2010): 263–272, 10.1378/chest.09-1584. 19762550

[23] J. Hur , Y. J. Kim , H. J. Lee , et al., “Cardioembolic Stroke: Dual‐Energy Cardiac CT for Differentiation of Left Atrial Appendage Thrombus and Circulatory Stasis,” Radiology 263, no. 3 (2012): 688–695, 10.1148/radiol.12111691. 22495682

[24] J. Hur , Y. J. Kim , H. J. Lee , et al., “Cardiac Computed Tomographic Angiography for Detection of Cardiac Sources of Embolism in Stroke Patients,” Stroke 40, no. 6 (2009): 2073–2078, 10.1161/STROKEAHA.108.533562. 19372451

[25] X. N. Li , J. X. Wang , Q. Wei , et al., “Diagnostic Value of Delayed Contrast‐Enhanced Cardiac Computed Tomography for Detecting Left Atrial Appendage Thrombus in Patients With Atrial Fibrillation,” Frontiers in Cardiovascular Medicine 9 (2022): 847163, 10.3389/FCVM.2022.847163. 35571218 PMC9095922

[26] D. Won , J. Walker , R. Horowitz , S. Bharadwaj , E. Carlton , and H. Gabriel , “Sound the Alarm: The Sonographer Shortage Is Echoing Across Healthcare,” Journal of Ultrasound in Medicine 43, no. 7 (2024): 1289–1301, 10.1002/JUM.16453. 38534218

[27] N. Yu , Y. Hong , X. Lv , Q. Liu , and M. Yan , “Preoperative Diagnostic Value of Multimodal Spectral CT for Patients With Atrial Fibrillation Undergoing Radiofrequency Ablation,” Frontiers in Medicine 11 (2024): 1440020, 10.3389/FMED.2024.1440020. 39328316 PMC11425045

[28] P. Karakasis , P. K. Vlachakis , P. Theofilis , et al., “Atrial Cardiomyopathy in Atrial Fibrillation: A Multimodal Diagnostic Framework,” Diagnostics 15, no. 10 (2025): 1207, 10.3390/diagnostics15101207. 40428200 PMC12110179

[29] M. Obed , M. M. Gabriel , E. Dumann , C. Vollmer Barbosa , K. Weißenborn , and B. M. W. Schmidt , “Risk of Acute Kidney Injury After Contrast‐Enhanced Computerized Tomography: A Systematic Review and Meta‐Analysis of 21 Propensity Score–Matched Cohort Studies,” European Radiology 32, no. 12 (2022): 8432–8442, 10.1007/S00330-022-08916-Y. 35727320 PMC9705469

[30] B. Choi , S. Heo , J. S. McDonald , et al., “Risk of Contrast‐Induced Acute Kidney Injury in Computed Tomography: A 16 Institutional Retrospective Cohort Study,” Investigative Radiology 60 (2025): 376, 10.1097/RLI.0000000000001141. 39602881

[31] W. Vandenberghe and E. Hoste , “Contrast‐Associated Acute Kidney Injury: Does It Really Exist, and If So, What to Do About It?,” F1000Research 8 (2019): 753, 10.12688/F1000RESEARCH.16347.1. PMC6544074 31275558

[32] B. J. W. Chow , M. Cheung , G. Prosperi‐Porta , et al., “Left Atrial Imaging Prior to Cardioversion,” JACC: Cardiovascular Imaging 17, no. 6 (2024): 702–704, 10.1016/J.JCMG.2023.12.009. 38363265

