# Supplementary material for: Safety and Feasibility of a CT‐Based Pathway for Left Atrial Appendage Assessment Prior to Inpatient Cardioversion: A Single‐Center Experience
Source: Clin Cardiol. 2026 Apr 27;49(5):e70323. doi: 10.1002/clc.70323 (PMC13112592; doi:10.1002/clc.70323)
Supplement: Supplementary file 1 — Supporting File 1 [file CLC-49-e70323-s001.docx]

**Supplemental Table S1.** Comparison of Baseline Patient Characteristics between Patients Who Underwent Pre-Cardioversion Imaging and Those Who Did Not (No-Imaging Group).

|  | **Overall (N=404)** | **No Imaging (N=124)** | **Imaging (N=280)** | **P value** |
| --- | --- | --- | --- | --- |
| **Demographics** | | | | |
| Median Age (IQR, 25%–75%) | 70.0 (62–76) | 71.2 (64–77) | 69.0 (61–76) | 0.09 |
| Sex (Males, N (%)) | 246 (60.9) | 72 (58.1) | 174 (62.1) | 0.51 |
| Median BMI (IQR, 25%–75%) | 28 (25–32) | 28 (26–32) | 28 (25–31) | 0.59 |
| **Comorbidities** | | | | |
| HTN (N (%)) | 269 (66.6) | 86 (69.4) | 183 (65.4) | 0.46 |
| DM (N (%)) | 124 (30.7) | 38 (30.6) | 86 (30.7) | 0.99 |
| Prior CVA\TIA (N (%)) | 41 (10.1) | 9 (7.3) | 32 (11.4) | 0.22 |
| Prior CKD (N (%)) | 68 (16.8) | 17 (13.7) | 51 (18.2) | 0.28 |
| **Thromboembolic Risk** | | | | |
| Median CHA₂DS₂-VASc (IQR, 25%–75%) | 3.0 (2–5) | 3.0 (1–4) | 3.0 (2–5) | 0.03 |
| CHA₂DS₂-VASc ≥2, N (%) | 316 (78.2) | 100 (80.6) | 216 (77.1) | 0.44 |
| **Anticoagulation** | | | | |
| Prior Anticoagulation (N (%)) | 401 (99.3) | 123 (99.2) | 278 (99.3) | 1.00 |
| **Duration of Anticoagulation Therapy** | | | | |
| Duration >30 days (N (%)) | 79 (19.6) | 18 (14.5) | 61 (21.8) | 0.09 |
| Duration 48h–30 days (N (%)) | 94 (23.3) | 25 (20.2) | 69 (24.6) | 0.35 |
| Duration <48h (N (%)) | 228 (56.4) | 80 (64.5) | 148 (52.9) | 0.03 |
| **Anticoagulation Type** | | | | |
| DOAC (N (%)) | 331 (81.9) | 111 (89.5) | 220 (78.6) | 0.01 |
| Dose – Standard (N (%)) | 280 (84.6) | 93 (83.8) | 187 (85.0) | 0.62 |
| Dose – Reduced/Adjusted (N (%)) | 51 (15.4) | 18 (16.2) | 33 (15.0) | — |
| VKA (N (%)) | 56 (13.9) | 8 (6.5) | 48 (17.1) | 0.001 |
| INR 2–3 (Therapeutic) | 28 (50.0) | 2 (25.0) | 26 (54.2) | 0.12 |
| INR outside 2–3 range | 28 (50.0) | 6 (75.0) | 22 (45.8) | — |
| Median INR (IQR, 25%–75%) | 2.40 (2.0–2.98) | 2.13 (1.22–2.91) | 2.41 (2.05–2.69) | 0.31 |
| Clexane (N (%)) | 14 (3.5) | 4 (3.2) | 10 (3.6) | 0.84 |

*Statistically significant differences (p<0.05) are highlighted in orange. The no-imaging group was characterized by significantly shorter anticoagulation duration prior to cardioversion (<48h: 64.5% vs 52.9%, p=0.03) and higher DOAC utilization (89.5% vs 78.6%, p=0.01), with lower VKA use (6.5% vs 17.1%, p=0.001), consistent with this group representing lower-risk cases managed per guideline recommendations for patients with documented therapeutic anticoagulation or appropriate short-duration DOAC use. The median CHA₂DS₂-VASc score was modestly lower in the no-imaging group (p=0.03), though the proportion with CHA₂DS₂-VASc ≥2 did not differ significantly (p=0.44).*

*BMI – body mass index; CKD – chronic kidney disease; CVA – cerebrovascular accident; DM – Diabetes Mellitus; DOAC – direct oral anticoagulant; HTN – Hypertension; TIA – transient ischemic event; VKA – vitamin K antagonist. CHA₂DS₂-VASc score calculated using the full standard nine-component algorithm (maximum score 9 points).*
