# Supplementary material for: Safety and Feasibility of a CT‐Based Pathway for Left Atrial Appendage Assessment Prior to Inpatient Cardioversion: A Single‐Center Experience
Source: Clin Cardiol. 2026 Apr 27;49(5):e70323. doi: 10.1002/clc.70323 (PMC13112592; doi:10.1002/clc.70323)
Supplement: Supplementary file 2 — Supporting File 2 [file CLC-49-e70323-s002.docx]

**Supplemental Table S2.** Anticoagulation Characteristics Prior to Cardioversion by Imaging Modality.

|  | **Overall (N=280)** | **CT-LAA (N=74)** | **TEE (N=206)** | **P value†** |
| --- | --- | --- | --- | --- |
| **Prior Anticoagulation** | | | | |
| Prior anticoagulation, N (%) | 278 (99.3) | 74 (100) | 204 (99.0) | 0.39 |
| **Duration of Anticoagulation Prior to Cardioversion** | | | | |
| >30 days, N (%) | 61 (21.8) | 11 (14.9) | 50 (24.3) | 0.11 |
| 48 hours – 30 days, N (%) | 69 (24.6) | 20 (27.0) | 49 (23.8) | 0.63 |
| <48 hours, N (%) | 148 (52.9) | 43 (58.1) | 105 (51.0) | 0.32 |
| **Direct Oral Anticoagulants (DOAC)** | | | | |
| DOAC, N (%) | 220 (78.6) | 64 (86.5) | 156 (75.7) | 0.06 |
| *Among DOAC recipients (CT n=64, TEE n=156):* | | | | |
| Standard dose, N (%) | 187 (85.0) | 57 (89.1) | 130 (83.3) | 0.31 |
| Reduced / adjusted dose, N (%) | 33 (15.0) | 7 (10.9) | 26 (16.7) | — |
| **Vitamin K Antagonist (VKA)** | | | | |
| VKA, N (%) | 48 (17.1) | 7 (9.5) | 41 (19.9) | 0.05 |
| *Among VKA recipients (CT n=7, TEE n=41):* | | | | |
| INR 2.0–3.0 (therapeutic), N (%) | 26 (54.2) | 2 (28.6) | 24 (58.5) | 0.22 |
| INR outside 2.0–3.0 range, N (%) | 22 (45.8) | 5 (71.4) | 17 (41.5) | — |
| Median INR (IQR, 25%–75%) | 2.41 (2.05–2.69) | 3.55 (2.18–4.01) | 2.41 (2.05–2.69) | 0.16 |
| **Low Molecular Weight Heparin** | | | | |
| Clexane (LMWH), N (%) | 10 (3.6) | 3 (4.1) | 7 (3.4) | 0.72 |

*CT-LAA – computed tomography aimed to visualize left atrial appendage; DOAC – direct oral anticoagulant; INR – international normalized ratio; LMWH – low molecular weight heparin; TEE – transesophageal echocardiography; VKA – vitamin K antagonist.*

*†All p-values derived from Fisher’s exact test, except median INR (Mann-Whitney U test). Duration categories are mutually exclusive. Dosing and INR subcategory p-values are calculated within the relevant subgroup (DOAC or VKA recipients). — indicates the complementary subcategory whose p-value is identical to the row above by construction and is therefore not repeated.*
